# A Comparison of the Development of Medical Informatics in China and That in Western Countries from 2008 to 2018: A Bibliometric Analysis of Official Journal Publications

**DOI:** 10.1155/2020/8822311

**Published:** 2020-10-12

**Authors:** Jun Liang, Zhongan Zhang, Lingye Fan, Dongxia Shen, Zhenying Chen, Jie Xu, Fangmin Ge, Junyi Xin, Jianbo Lei

**Affiliations:** ^1^IT Center, Second Affiliated Hospital, School of Medicine, Zhejiang University, Hangzhou, Zhejiang Province, China; ^2^Performance Management Department, Qingdao Central Hospital, Qingdao, Shandong Province, China; ^3^Department of Obstetrics and Gynecology, Affiliated Hospital, Southwest Medical University, Luzhou, China; ^4^Editorial Department of Journal of Practical Oncology, Second Affiliated Hospital, School of Medicine, Zhejiang University, Hangzhou, Zhejiang Province, China; ^5^Library of Zhejiang University, Hangzhou, Zhejiang Province, China; ^6^International Network Medical Center, Second Affiliated Hospital, School of Medicine, Zhejiang University, Hangzhou, Zhejiang Province, China; ^7^Hangzhou Medical College, Hangzhou, Zhejiang Province 310000, China; ^8^Institute of Medical Technology, Health Science Center, Peking University, Beijing, China; ^9^Center for Medical Informatics, Peking University, Beijing, China; ^10^School of Medical Informatics and Engineering, Southwest Medical University, Luzhou, Sichuan Province, China

## Abstract

**Objective:**

We focused on medical informatics journal publications rather than on conference proceedings by comparing and analyzing the data from journals and conferences from a broader perspective. The aim is to summarize the unique contributions of China to medical digitization and foster more multilevel international cooperation.

**Method:**

In February 2019, publications from 2008 to 2018 in three major English-language medical informatics journals were retrieved through Scopus, including the journals, namely, International Journal of Medical Informatics (IJMI, international community), JAMIA (United States), and Methods of Information in Medicine (MIM, Europe). Three major Chinese-language journals, namely, China Digital Medicine (CDM), Chinese Journal of Health Informatics and Management (CJHIM), and Chinese Journal of Medical Library and Information Science (CJMLIS), were searched within the major three Chinese literature databases. The datasets were preprocessed using the NLP package on Python, and a smart local moving algorithm was used as a clustering method for identifying the aforementioned journals.

**Result:**

Between 2008 and 2018, the total number of published papers and H-index of the three English-language journals was 1371 and 67 (IJMI), 1752 and 86 (JAMIA), and 637 and 35 (MIM), respectively. In the same period, the total number of published papers and H-index in the three Chinese-language journals was 6668 and 23 (CDM), 1668 and 22 (CJHIM), and 2557 and 25 (CJMLIS), respectively. IJMI, JAMIA, and MIM received submissions from 82, 59, and 62 countries/regions, respectively. By contrast, the three Chinese journals only received submissions from seven foreign countries. The proportions of authors from institutional affiliations were similar between the three English-language journals (IJMI, JAMIA, and MIM) and CJMLIS because the majority of the authors were from universities (81%, 74%, 73%, and 65.2%), followed by medical institutions (12%, 10%, 9%, and 23.4%) or research institutes (2%, 4%, 10%, and 4.3%). Furthermore, the proportions of the authors from enterprises were low (2%, 6%, 4%, and 0.3%) for all journals. However, the authors in CDM and CJHIM were mainly from medical institutions (50% and 40%), followed by universities (33% and 32%) and research institutes (3% and 4%). In addition, the proportions of enterprises were only 3% and 2%, respectively. Among the top five authors in three English-language journals (ranked in terms of the number of published papers), 100% had doctoral or master's degrees, compared with only 60% in the Chinese journals. Additionally, 28204 different keywords were extracted from the aforementioned papers, covering 275 specific high-frequency key terms. Based on these key terms, four clusters were found in the English literature—“Health and Clinical Information Systems,” “Internet and Telemedicine,” “Medical Data Statistical Analysis,” and “EHRs and Information Management”—and three clusters were found in the Chinese literature: “Hospital Information Systems and EMR,” “Library Science and Bibliometrics Analysis,” and “Medical Reform Policy and Health Digitization.” Only two clusters are similar, and Chinese-language journals focus more on health information in technology and industrial applications than in medical informatics basic research.

**Conclusion:**

This study provides important insights into the development of medical informatics (MI) in China and Western countries showing that the medical informatics journals of China, the United States, and Europe have distinct characteristics. Specifically, first, compared with the Western journals, the number of papers published in the journals of professional associations in the field of MI in China is large and the application value is high, but the academic influence and academic value are relatively low; second, most of the authors of the Chinese papers are from hospitals, and most of the counterparts in the Western countries are from universities. The proportion of master's or doctoral degrees in the former is also lower than that of the latter; furthermore, regarding paper themes, on the one hand, China MI has no theoretical and basic research on medical data statistics and consumer health based on the Internet and telemedicine; on the other hand, after nearly 10 years of hospital digital development, China has fully used the latecomer and application advantages in hospitals and, through extensive international cooperation, has made significant advancements in and contributions to the development of medical information.

## 1. Introduction

Medical informatics (MI) can be defined as the acquiring, storing, retrieving, and using of healthcare information to foster better collaboration among a patient's various healthcare providers [1], which originated in 1959 when Ledley et al. published “Reasoning Foundations of Medical Diagnosis; Symbolic Logic, Probability and Value Theory Aid Our Understanding of How Physicians Reason” in Science [2]. Increasing MI is a fundamental requirement for building effective and efficient health information systems at the local, national, and global levels [3]. With the increasingly extensive application of computer science and information technology in medical fields, MI has gradually become an interdisciplinary field theoretically based on computer technology and science that integrates medicine and information science and management to achieve digitized medical management at the global level [3].

The establishment and development of a discipline rely on the foundation and support of a system, including relevant institutions and associations, mainstream auxiliary journals, and publications [4], for which there is no exception for MI. Most developed nations have all established corresponding disciplinary systems, including institutes, conferences, and journals, and have actively developed talent, culture, and scientific research. IMIA was founded in 1978, is the acknowledged leader of international MI, and comprises over 45 state-level organization institutes and four regional conferences. IMIA holds one MI academic conference, MedInfo, biannually. IMIA has four official journals, which are all included in the Science Citation Index (SCI), namely, the International Journal of Medical Informatics (IJMI), Applied Clinical Informatics, Informatics for Health and Social Care, and Methods of Information in Medicine (MIM). Of these four journals, IJMI is the most influential. The European Federation for Medical Informatics (EFMI), established in 1976, is one of four regional conferences of the IMIA, comprises 30 state-level institutes, and is mainly devoted to exchanges among European countries. It holds its MI academic conference (MIE) annually. EFMI has four official journals, of which two are included in the SCI, namely, MIM (founded in 1962) and IJMI. AMIA is the official representative institution of the United States in IMIA and was jointly founded from three organizations in 1990. Its members are not limited to the United States and are from interdisciplinary organizations across 65 countries, including those of doctors, nurses, engineers, medical librarians, institute researchers, and educators. AMIA holds annual conferences, and its official journal, *Journal of the American Association* (JAMIA), is also included in the SCI.

Different from MI in Western countries that developed from computer applications to medicine, MI in China evolved from medical library information science in the early 1980s. Along with the development of hospital digitization in China, this discipline did not become independent until 2010. To date, only a few educational institutions in China have established an MI institute or graduate courses (27 programs for a master's degree, five programs for a doctorate), and the majority of participants are undergraduates [5]. Furthermore, China currently has four state-level medical information associations, of which three have journals: China Digital Medicine (CDM) from the China Hospital Information Management Association (CHIMA), Chinese Journal of Medical Library and Information Science (CJMLIS) from the Chinese Society of Medical Information (CSMI) (founded by Chinese Medical Association), and Chinese Journal of Health Informatics and Management (CJHIM) from the Chinese Health Information Association (CHIA) (formerly known as the Chinese Health Statistics Association).

To efficiently, accurately, and timely understand the research hot spots and developing trends of medical informatics, researchers have adopted bibliometrics to study the frontiers of MI from three aspects: academies, conferences, and journals. As for academies, V. Maojo et al. in 2012 reviewed the members attending three mainstream academies of MI, including Medical Informatics Europe (MIE) 2005–2008, MedInfo 2004, 2007, and 2010, and AMIA 2005–2009, and thought that the influence of these academies outside this discipline was very low [6]. As for conferences, Liang et al. compared the characteristics between Chinese and international mainstream MI conferences from the aspects of conference history, scales, and themes [7]. Jia et al. comprehensively analyzed the authors, academic values, and themes between Chinese and international mainstream MI conference publications [8]. Moreover, as for journals, by using online bibliographic search filters, Van Kasteren. et al. analyzed the abstracts, titles, and keywords among JMIR, MIM, JAMIA, and IJMI in 2001–2015 and analyzed and summarized the changing trends of themes in these English-language MI journals [9]. With UCINET, NetDraw, and SPSS, Deng et al. analyzed 1340 papers published by Chinese academics in 18 journals listed in the 2016 JCR under the MI category, plotted keyword cluster trees and cooccurrence network diagrams, and found that the research hot spots of Chinese-language journals were MI systems, mobile healthcare, and telecare [5]. Kim and Delen analyzed the themes of 26407 English-language papers published in 23 journals listed in the 2012 JCR under the MI category during 2002–2013 by using keyword clustering and found that the research hotspots and mainstreams in MI were HIT, Internet-enabled research, and EMR/EHRs [10]. Gukesen and Haux analyzed the theme trends of English-language papers published in 23 journals listed in the 2016 JCR under the MI category during 2013–2017 using VOSviewer, and they recognized 5 theme clusters, including biomedical data analysis and clinical informatics [11]. However, the existing studies are quantitative analyses of medical informatics research hotspots performed internationally or in China, but there is rare comprehensive comparison of the two, and the existing comparisons are limited by the nonunification of time dimensions. Our study is based on the overall research framework of our previous studies, or, namely, the principle that this discipline cannot be developed without the support from professional academies, mainstream conferences, and journals. We have expanded and added the comprehensive analysis of MI academic journals in China, the United States, and Europe and from the international MI community (IMIA) to find gaps in Chinese MI. We then summarized the shared experiences with other countries to promote international exchange and help improve and develop Chinese MI. Our research filled the gap in comprehensive comparative analysis and had unique contributions.

## 2. Materials and Methods

### 2.1. Journal Sources and Selection Criteria

Under the framework of Preferred Reporting Items for Systematic Reviews and Meta-Analysis (PRISMA) [12], We selected three representative English-language MI association journals—International Journal of Medical Informatics (IJMI: IMIA, global), Journal of the American Medical Informatics Association (JAMIA: AMIA, United States), and MIM (EFMI, Europe)—that represent different research groups and regions. For the Chinese-language MI journals, we selected three representative journals and the largest-scale professional journal from state-level MI associations: the official journal of CHIMA: CDM (CHIMA, China); CJHIM (CHIA, China) from CHIA; and the CJMLIS (CSMI, China) from the Medical Information Subassociation.

### 2.2. Data Collection and Preprocessing

First, the English-language papers were searched on Scopus, which provided comprehensive preprocessed data from academic publications and has been accepted as one of the best databases for literature analysis [13]. The search period was limited to 2008–2018, and the literature types searched for were articles and reviews. Second, the Chinese-language papers were searched through three major Chinese literature databases: Chinese Science and Technology Journal Database (CQVIP) [14], Chinese National Knowledge Infrastructure (CNKI) [15], and WanFang Data [16]. EndNote X7 was used for a preliminary analysis of the general information assembled since it is able to not only merge and filter bibliographic records in different formats but also exclude some articles such as book reviews, conference notifications, and those which are not related to MI. The searching period was from 2008 to 2018, and to ensure data consistency, all searches were conducted on February 7, 2019.

Because all the databases had standard functions for data analysis and abstraction, we selected and imported the following information:

1. Metadata of journals, including the names of journals, organization of the sponsors, organization of the publication, time of publication, period of publication, place of publication, and database of inclusion
2. Metadata and contents of periodical papers, including the title; keywords; abstract; year of publication; citations of papers; and authors' names and education level, affiliations, and country

Next, to maximize the effectiveness and accuracy of co-word analysis and data visualization, we preprocessed the target datasets. First, the titles and keywords of the included articles were standardized into lowercase, and the morphology and abbreviations were reproduced in Python 2.7 + NLTK NLP [17]. Second, because all the Chinese-language MI periodical papers were written in Chinese, we used the self-developed Chinese Latin tool to convert the Chinese names into Pinyin. Pinyin (phonetic alphabet) is a system of romanization of Chinese characters and represents the pronunciation of Chinese characters. Pinyin was approved in 1958 by the government of the People's Republic of China and officially adopted in 1979. Pinyin is not used officially in Taiwan [18]. Furthermore, using the Youdao AICloud translation package [19], we translated the titles and keywords into English and manually emended and examined them. Finally, these preprocessed datasets of periodical papers were collected and imported into Microsoft Excel 2011 and EndNote X2 for further qualitative and quantitative analyses.

### 2.3. Bibliometric Analysis

**Figure 1:**
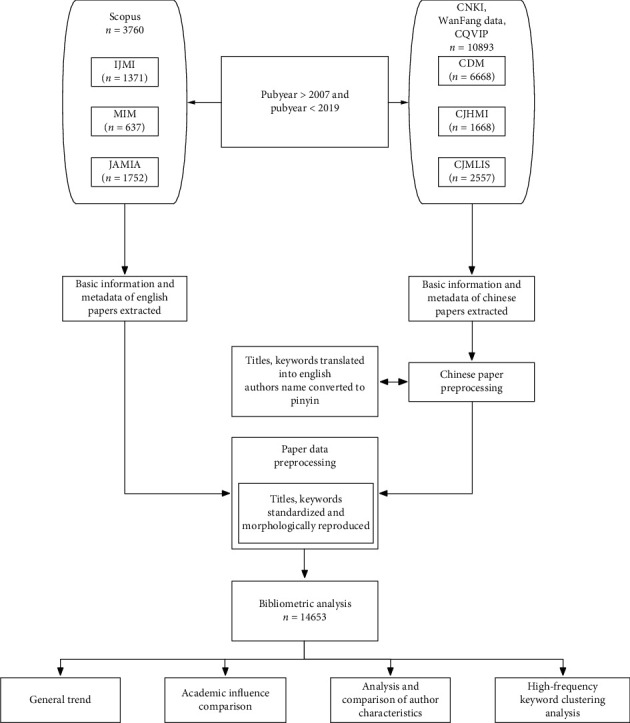
Workflow of the target article search and major manipulations.

The basic characteristics of the papers were analyzed with the built-in functions of Scopus, CQVIP, CNKI, and WanFang Data. The author affiliations were clustered using self-developed tool based on Python 2.7. The tool clusters the authors' affiliations into six categories: medical institutions, universities, manufacturers, research institutions, and others. The H-index was designed as a measure of scientific research impact [20], which indicates that a scholar or country has published H papers, and each of which has been cited in other publications at least H times. Therefore, the H-index reflects both the number of publications and the number of citations per publication [21]. Co-word analysis was proposed by Michel and Jean-Pierre from the France National Centre for Scientific Research [22]. In this case, on the one hand, we adopted a smart local moving algorithm [23] as the word clustering method; on the other hand, the results of clustering were visualized on VOSviewer (Centre for Science and Technology Studies, Leiden, Netherlands) [24]. The process is illustrated in Figure 1.

## 3. Results

### 3.1. Overall Trends of Journal Papers

Papers published in MI journals between 2008 and 2018, including the three Chinese MI journals (CDM, CJHIM, and CJMLIS) and the three English MI journals (IJMI, MIM, and JAMIA), were analyzed both quantitatively and qualitatively. The basic information of each journal is listed in Supplementary Materials Table S1. Since CDM was founded in 2007, we selected papers published after 2008, which facilitated a comparison between different MI journals.

**Figure 2:**
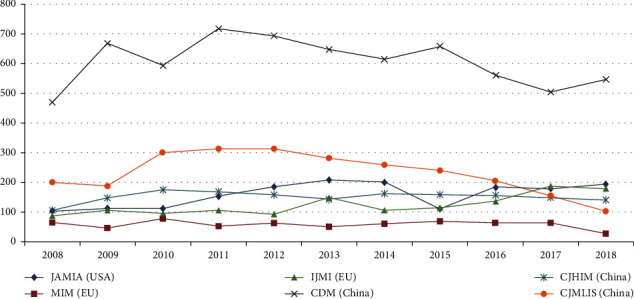
Journal articles of the IJMI, JAMIA, MIM, CDM, CJHIM, and CJMLIS from 2008 to 2018.

From the three mainstream Chinese-language databases, 6668, 1668, and 2557 articles between 2008 and 2018 were identified from CDM, CJHIM, and CJMLIS, respectively, with a total of 10893 and an annual rate of 990 articles. Using Scopus, 1371, 637, and 1752 articles were identified from IJMI, MIM, and JAMIA, respectively, with a total of 3760 and an annual rate of 341 articles. The number of Chinese-language papers was 2.89 times that of English-language papers, and the number of papers in CDM was 1.77 times that of the total number of English-language papers (Figure 2). The proceedings from Chinese mainstream MI association conferences, including the China Conference (CMIAAS), China Hospital Information Network Conference (CHINC), Chinese Health Information Technology Exchange Conference (CHITEC), and Chinese Medical Association National Medical Information Conference (CPMI), had no unified search database and were not continuous or complete [8, 25]. However, the papers published in CDM, CJHIM, and CJMLIS could be completely and timely retrieved from the three mainstream Chinese-language databases. These phenomena all suggest Chinese MI journals as the main disciplinary systems that support the establishment and development of the MI discipline in China.

### 3.2. Academic Influence of Professional Journals

**Figure 3:**
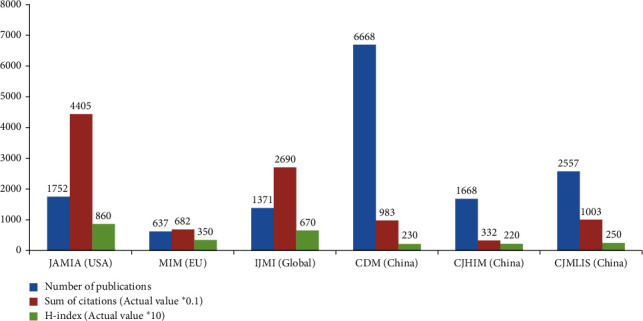
Number of publications, citations, and H-indices of IJMI, JAMIA, MIM, CDM, CJHIM, and CJMLIS in 2008–2018.

The academic influence of the professional MI journals is significantly different. JAMIA and IJMI are top-ranked journals, followed by MIM; however, the Chinese MI journals (CDM, CJHIM, CJMLIS) are ranked much lower. JAMIA published 1752 papers between 2008 and 2018, which were cited 44051 times, with an average of 25.1 times per paper and an H-index of 86 (Figure 3). IJMI published 1371 papers in this period, which were cited 26900 times, with an average of 19.6 times per paper and an H-index of 67. MIM published 637 papers in this period, which were cited 6827 times, with an average of 10.7 times per paper and an H-index of 35. The aforementioned three journals can all be retrieved on SCI, and the 2017 impact factors of JAMIA, IJMI, and MIM were 4.27, 2.975, and 1.531, respectively.

We compared similar statistics from the three Chinese MI journals, and the results suggest that the total citations of the three Chinese MI journals are slightly lower than that of IJMI (23184 vs. 26900) but far lower than that of JAMIA (44051), even though the total number of papers published by the three Chinese journals was 16.9 and 13.2 times those in IJMI and JAMIA, respectively.

### 3.3. Author Analysis of MI Journals

#### 3.3.1. Author Distributions of Journal Papers

**Figure 4:**
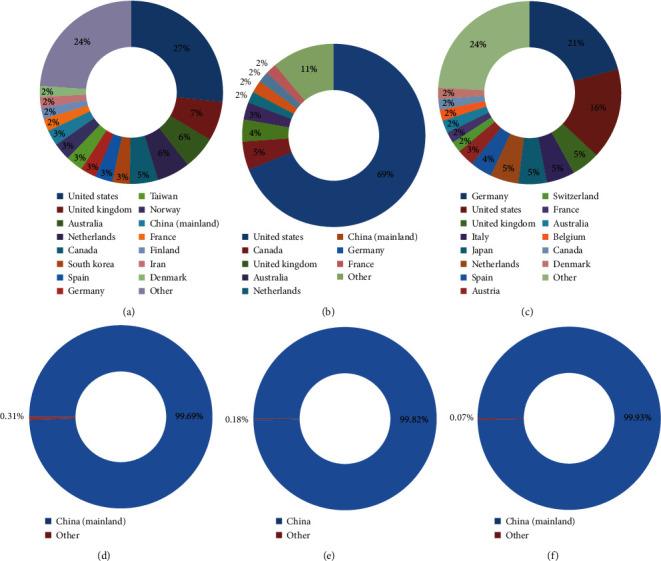
Analysis of author distribution among the countries in (a) IJMI (EU), (b) JAMIA (USA), (c) MIM (EU), (d) CDM (China), (e) CJHIM (China), and (f) CJMLIS (China) in 2008–2018.

Similar to the country distribution of authors in the MI conference proceedings, the regional MI journals, including JAMIA (AMIA, United States) and MIM (EFMI, Europe), and Chinese MI journals (CDM, CJHIM, and CJMLIS) were dominated by local authors, whereas the international journals such as IJMI (IMIA, global) represented authors from various regions. Between 2008 and 2018, JAMIA published submissions from authors of 58 countries or regions, and the number of countries or regions with >10 manuscripts was 15; however, the majority of authors were from the United States (approximately 69%), followed by Europe (∼10%). By contrast, the proportion of the authors from China (including mainland China, Taiwan, Hong Kong, Macao) was only 4%. MIM published submissions of the authors from 62 countries or regions, and the number of countries or regions with >10 manuscripts were 21. However, most authors were from Europe (approximately 63%), followed by the United States (about 16%), and the proportion of authors from China (including mainland China, Taiwan, Hong Kong, Macao) was only 2.4%. IJMI published submissions from the authors of 81 countries or regions; the number of countries or regions with >10 manuscripts was 28; and approximately 27%, 40%, and 6% of authors were from the United States, Europe, and China (including mainland China, Taiwan, Hong Kong, Macao), respectively. The Chinese MI journals (CDM, CJHIM, CJMLIS) were less internationalized because publications by foreign authors were only from Japan, Canada, the United States, South Korea, the United Kingdom, Netherlands, and Germany.  Figure 4 shows the countries with more than 1% of the authors.

#### 3.3.2. Author Characteristics of Journal Papers

**Figure 5:**
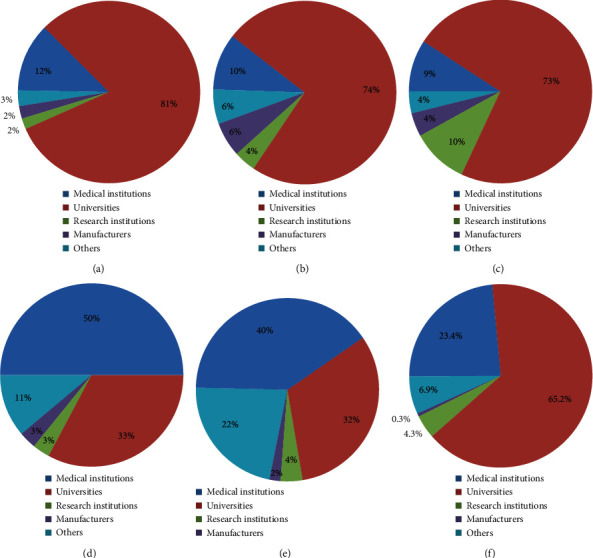
Author affiliations in (a) IJMI (EU), (b) JAMIA (USA), (c) MIM (EU), (d) CDM (China), (e) CJHIM (China), and (f) CJMLIS (China) in 2008–2018.

The affiliations and academic backgrounds of the authors were largely different among journals. The proportions of different affiliations were similar between the English-language journals (IJMI, JAMIA, MIM) and the Chinese journal CJMLIS because the majority (>50%) of authors were from universities (81%, 74%, 73%, and 65.2%, respectively), followed by medical institutions (12%, 10%, 9%, and 23.4%, respectively) or research institutes (2%, 4%, 10%, and 4.3%, respectively). Furthermore, the proportions of enterprises were low (only 2%, 6%, 4%, and 0.3%, respectively) across all journals. However, the authors in CDM and CJHIM were mainly from medical institutions (50% and 40%, respectively), followed by universities (33% and 32%, respectively) and research institutes (3% and 4%, respectively), and the proportions of enterprises were only 3% and 2%, respectively (Figure 5). These data are very similar to the affiliation distributions in the international MI conference proceedings; however, they differ from those in the Chinese MI conference proceedings because the latter is dominated by medical institutions (54%), followed by universities (17%), institutes (10%), and enterprises (7%) [25].

We further assessed the top five authors in each journal and analyzed their affiliations and academic and knowledge backgrounds (Supplementary Materials Table S2). We observed that the authors of JAMIA and MIM differed from those of the other journals but were similar to the authors from the proceedings of AMIA, MIE, and MedInfo because a large proportion (40%) of authors possessed MD degrees [8].

The top five authors in IJMI had academic backgrounds similar to those in the Chinese MI journals (CDM, CJHIM, CJMLIS) and Chinese MI academic journals (CMIAAS, CHINC, CHITEC, CPMI) because the majority of authors had no background in clinical medicine. Of the top five authors in IJMI, one had an MD, four had doctoral degrees, and all were from universities. Among the top five authors in CDM, one had a master's and doctoral degree, three had doctoral degrees, and two had master's degrees. Furthermore, two were from medical institutions and three were from institutes.

We also observed some differences because the top five authors from IJMI, MIM, and JAMIA all received professional academic training in MI; however, the top five authors in the Chinese MI academic journals mostly did not receive this training; usually were from the computer, public health, statistics, library science, or informatics fields; and only became involved in MI after employment.

### 3.4. Keyword Selection and Analysis of Journal Papers

The purpose of the keyword selection and analysis is to identify the focal points of research that have been confirmed as a major step in monitoring the development and trend of a discipline [26]. Here, the keywords in the papers published in the English-language journals (IJMI, MIM, JAMIA) and Chinese-language journals (CDM, CJHIM, CJMLIS) were analyzed and compared to determine the underlying rules.

#### 3.4.1. Selection of High-Frequency Threshold of Keywords

The frequency distributions of the keywords were largely different between the Chinese- and English-language MI journals, which led to differences in the high-frequency word selection methods. To simplify the selection and analytical process and to decrease unnecessary interference from low-frequency words, we selected high-frequency words. However, no unified method is available to determine the critical levels of high-frequency words, and the available methods include the subjective empirical method, 80/20 rule [27], Price's equation [28], *g*-index [29], and high/low-frequency word isolation equation.

To select the appropriate frequency threshold, we first observed the frequency distributions of keywords in the six MI journals and found that the repeated rates of keywords were low in papers from Chinese MI journals compared with those in the international high-quality and mainstream MI journals (IJMI, MIM, and JAMIA). Namely, many keywords occurred once, and the rate of these words was 20% higher than those in the English-language papers, which limited the applicability of the high/low-frequency word isolation method based on Zipf's law. Specifically, the threshold value was slightly larger, and too few high-frequency words were intercepted. After multiple trials, we adopted different high-frequency word threshold computation methods according to the characteristics of English-language and Chinese-language papers and used Donohue's method [30] based on Zipf's law for the English papers. The formula is as follows:

(1) $$\begin{array}{c} T=\frac{\sqrt{1+8\;\times N_{1}}-1}{2}\;, \end{array}$$

where *N*_1_ is the number of keywords with one word frequency and *T* is the frequency threshold of high-frequency words.

For the Chinese papers, according to the 80/20 rule [27], high-frequency words accounting for an accumulated proportion of 20% were extracted in a descending manner. The final high-frequency word frequency thresholds were 112 for the English-language journals and 26 for the Chinese-language journals.

#### 3.4.2. High-Frequency Keyword Clustering Analysis and Research Focal Points

Analysis of keywords can indirectly reveal the hotspots and changing trends in research topics, which is critical for understanding the development of this field [31]. Next, we mined the data in the keywords in the published articles from the two groups of six MI journals. First, 28204 keywords were identified from 14653 articles. Second, these keywords were filtered by using the aforementioned high-frequency word thresholds. Next, the keywords with semantic similarity or closeness were grouped by using the “replace by” column on VOSviewer. We found 275 highly correlated high-frequency words: 153 words from the Chinese-language journals and 132 words from the English-language journals. Finally, the keywords in the two groups were clustered.

The results showed the keywords from the English-language MI journals were reorganized into four clusters, which we named (1) “Internet and Telemedicine” (yellow), (2) “Health and Clinical Information Systems” (blue), (3) “Medical Data Statistical Analysis” (green), and (4) “EHRs and Information Management” (red). Notably, our clustering results were supported by Kim et al., who mined the abstracts and texts from the articles published in 23 English-language MI journals within 12 years [10]. Only three clusters were found in the Chinese-language MI journals and were named (1) “Hospital Information Systems and EMR” (red), (2) “Library Science and Bibliometrics Analysis” (purple), and (3) “Medical Reform Policy and Health Digitization” (orange).

**Figure 6:**
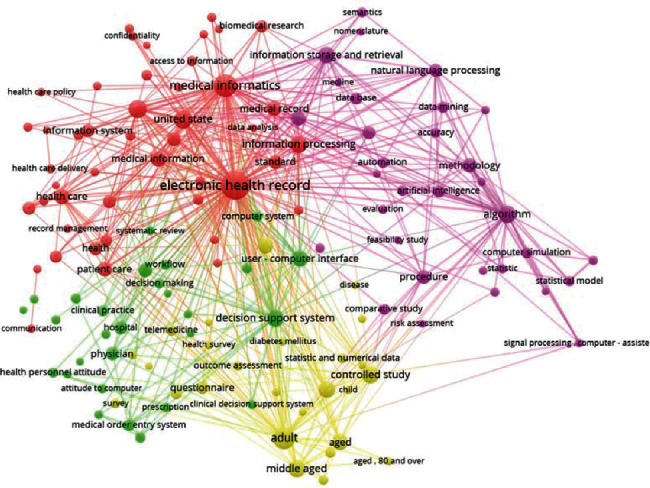
Cluster map of the keywords obtained by text mining 11  years (2008–2018) of articles on different topics from three English MI journals (IJMI, JAMIA and MIM). Topics are colored as red (EHRs and Information Management), blue (Health and Clinical Information Systems), yellow (Internet and Telemedicine), and green (Medical Data Statistical Analysis).

**Figure 7:**
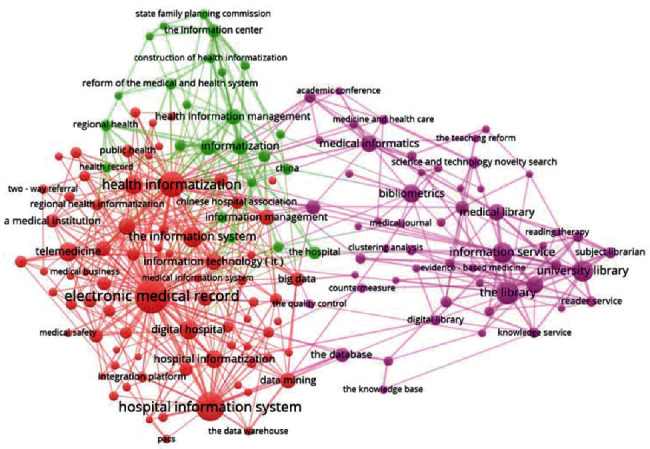
Cluster map of the keywords obtained by text mining 11  years (2008–2018) of articles on different topics from three Chinese MI journals (CDM, CJHIM, and CJMLIS). Topics are colored as red (Hospital Information Systems and EMR), orange (Medical Reform Policy and Health Digitization), and purple (Library Science and Bibliometrics Analysis).

The results of the clustering are presented in Figures 6 and 7. Moreover, the ten keywords with the highest frequency in each cluster are listed in Table 1. Due to length limitations, a discussion of the concerns of the six journals is in Supplementary Materials Content S3.

## 4. Discussion

We previously analyzed the MI conference proceedings in China, the United States, Europe, and IMIA and found that MI research in China was largely different and lagged behind other developed regions in terms of academic evaluation, multisource cooperation, talent pool and quality, focal points, trends, and research investment [8]. In this study, we further comprehensively analyzed the data from MI journals to confirm our previous findings from a broader perspective and thereby propose key recommendations.

### 4.1. Analysis and Comparison of Academic Values between Chinese and English MI Journals

MI as an emerging interdisciplinary field has not been set within a specific category in the WOS but has a similar category—Medical Informatics—involving 25 journals. JAMIA (the 2017 impact factor: 4.27), IJMI (the 2017 impact factor: 2.95), and MIM (the 2017 impact factor: 1.53) rank as 3, 6, and 17, respectively. In particular, JAMIA has been highly approved by the Academic Committee of China Computer Society, listed by the China Computer Society as a key recommendation, is a well-known and highly reputed journal in the cross-disciplinary/comprehensive/emerging field, and encourages submissions from Chinese counterparts [32].

However, China has no authorized MI journal or any MI journal included in SCI, EI, or Medline. We believe that this may be related to the academic levels of these journals. Of the three authorized MI professional journals, the H-indices of CDM, CJHIM, and CJMLIS are 23, 22, and 25, respectively, which are less than half that of IJMI and are 70% that of MIM. However, they are slightly higher than that of MEI and the MedInfo proceedings (H-index of both: 19) and lower than that of the AMIA Annual Symposium (H-index: 32) [8]. Furthermore, the check and encouragement criteria in Chinese academia are first based on the articles included in SCI, Ei engineering village (Ei), or Medline and then on the core journals included in the Peking University Core Journals List, which are primarily graduate student dissertations [4]. However, none of CDM, CJHIM, or CJMLIS was included in the Chinese Core Journals List [33]. Thus, many researchers can only submit to nonprofessional MI Chinese core journals, which increases difficulty and limits dissemination. This phenomenon of no core journals or high-quality journals and no high-influence or high-cited journals severely constrains the development of mainstream journals and publications that support MI.

### 4.2. Analysis and Comparison of Regional Cooperation and Author Characteristics between Chinese and English MI Journals

IJMI, MIM, and JAMIA, as authorized academic journals in MI, have attracted submissions from academic researchers and industrial workers worldwide; however, the Chinese MI journals (CDM, CJHIM, and CJMLIS) have received submissions from only seven foreign countries. However, in the IJMI, MIM, and JAMIA journals, the proportion of Chinese authors (including mainland China, Taiwan, Hong Kong) did not exceed 6%, which implies gaps in MI development in China.

China has an insufficient talent pool for MI and has no continued support from existing educational institutions for the advancement of industrial development, which is stated as “cold in academic research, (and) incorporate(s) hot in industrial application” [4]. The author affiliations in CDM and CJHIM are mainly medical institutions, whereas the majority of those in JAMIA, IJMI, MIM, and CJMLIS are universities. A much larger proportion of authors have doctoral degrees and a medical educational background in IJMI, MIM, and JAMIA compared with the qualifications of the authors in the seven Chinese MI academic journals.

Such a difference in author characteristics between the Chinese journals and journals from other regions is because of the distinguished developmental environment for MI in China. In 2009 in China, HIT was considered one of the “ four beams and eight pillars” of the new healthcare reform [34], and MI was treated as an independent discipline. However, MI was equated as HIT to a large extent. In third-class hospitals (the highest class), the IT workers mostly undergo undergraduate courses or below; therefore, submissions are mainly summarized in the context of hospitals but ignore theories of MI, especially research on methodology and basic technology.

### 4.3. MI Journals from China and Western Countries: Analysis and Comparison in Keyword Clusters

In Figures 6 and 7, seven keyword clusters were identified from the six Chinese-language or English-language MI journals; of them, two clusters (EN-Cluster 2 and CN-Cluster 1) are similar, which again reflects “hot in industry application, and cold in academic research” in the MI field of China. Next, we discuss these seven clusters:

1. EN-Cluster 1 (“Internet and Telemedicine”): this cluster results from the introduction of the Internet into the medicine field and is based on extensive research on diabetes and family care through the Internet, telemedicine, and other techniques and through questionnaires. Surprisingly, telemedicine in Europe and the United States has formed an independent system, but in China telemedicine belongs to the cluster “Hospital Information Systems and EMR” and is an important part of hospital information systems (HIS) and a supplement to out-hospital continuity of medical services to patients. We posit that this situation may be related to the “digital hospital” policies in China. On the one hand, in the *EMR Function Grade Specification* issued by the National Health Commission of China in 2015, telemedicine was already included in EMR [35]; on the other hand, our preliminary research implied that the Chinese government has involved telemedicine as a major part of regional HIT construction and has achieved great success [36].
2. EN-Cluster 2 (“Clinical and HIS”) and CN-Cluster 1 (“HIS and EMR”): clinical informatics [37], especially the use of computers in hospital management, clinical diagnosis, and treatment, is the focus of global researchers and industrial workers in this field, including medical order entry systems, electronic medical records, and other HIS. We think that the reason may be that HIS have been extensively applied in hospitals [38] and that HIT has been gradually approved by the medical field [39]. Moreover, along with the deepening of medical reforms in China in recent years, the National Health Commission of China has expanded the connotations of Chinese HIS and proposed the goal of “digital hospitals” [40]. A notable requirement is that hospitals are starting to expand to digitization services, including patient engagement services (e.g., schedule appointments online; pay bills online; view, download, and print their medical information; and participate in satisfaction evaluations).
3. EN-Cluster 3 (Medical Data Statistical Analysis): this cluster is exclusive to the English-language MI journals and involves abundant content related to theoretical models, including the topics associated with medical information methodology, artificial intelligence, natural semantic processing, and data mining software and systems.
4. EN-Cluster 4 (EHRs and Information Management): this cluster is also exclusive to the English-language MI journals and describes an important aspect of MI. We posit that this also means that EHRs have gradually become a widely approved and accepted concept. The European and US researchers are focusing on technical issues of EHRs. Notably, EHRs are not present in the high-frequency keyword list in China; nevertheless, its in-hospital version—EMR [41]—is the core of “HIS and EMR” and is the keyword with the highest frequency in CN-Cluster 1. Effective use of the data of EMR/EHRs is necessary for the development of evidence-based medicine [42], but, compared with Europe and the United States with their well-developed MI, the mining, analysis, and use of data of EMR/EHRs in China remain in the early stage. Nevertheless, medical reforms in China, especially reforms of health insurance payment systems (e.g., China has released medical insurance reimbursement policies based on China Healthcare Security Diagnosis Related Groups and has gradually transited from the conventional “pay-for-service” to “pay-for-performance” system [43]), have deepened, and AI techniques [44] and the importance of medical data (China included medical big data as a national strategic resource in 2016 [45]) have been largely improved. Given these advances, we posit that the exploitation of medical data of EMR/EHRs at the level of scientific development, the improvement in medical quality, and the decrease in medical errors and medical expenses will all soon become the foci and hot spots of MI research in China. With the rapid development and wide application of wearable device technology [46, 47], more real-time health data can be included in EHRs, which further promotes this trend.
5. CN-Cluster 2 (Library Science and Bibliometrics Analysis): this cluster is exclusive to the Chinese-language MI journals, and its research and application are concentrated in medical information research; medical information resources construction, retrieval, and services; and other health information management and evaluations. We posit that this occurs because the development of MI in China originated from library science in the mid-1980s, and this narrow sense of MI continues to account for a large proportion in China.
6. CN-Cluster 3 (Medical Reform Policy and Health digitization): this cluster is exclusive to China and involves the propaganda, interpretation, analysis, and comments of HIT policies issued by healthcare reforms and government functional departments.

### 4.4. Comparative Analysis of Medical Information Education Systems

As an independent discipline, MI has been widely accepted in Europe and the United States. To date, greater than 80 US-based academic institutions offer on-site or distance MI training programs [48]. Additionally, established universities in Western countries have all established the major of medical information, mostly in master and doctorate education, and focus on computer technology and methods, association with clinical activities, and practicality. By contrast, China established an MI-related major in 1983, which was gradually developed from the library information major in medical universities. To date, in China, education in this field mainly comprises undergraduate programs and only has 27 master's programs and five doctoral programs [4], and these numbers are less than half of those of the United States. The education focuses on information management theories and methods but rarely focuses on the technical application of computers; thus, the practical operation abilities of students are low. Consequently, the MI academy of China cannot recruit a sufficient number of qualified applied talents to the industry, and the industry does not highly evaluate the quality of these talents.

### 4.5. Implications of This Study

This study provides notable contributions to theory and practice.

From the theoretical perspective, first, this study further enriches and improves the knowledge of global MI literature research; in particular, for the first time, this study adds the analysis of Chinese MI journal literature. On this basis, our comparison of bibliometrics between three representative official English journals of the international MI societies and three Chinese journals of the national MI societies overcomes the limitation that some bibliometrics research in the field of MI has mainly focused on English-language literature [10, 42, 48].

Second, we explore the differences in authors between those in China and Western countries in their organizations and academic degrees. We find that on the one hand, most of the authors of Chinese papers are from medical institutions, and most of their counterparts in Western countries are from universities. The proportion of doctoral or master's degrees in the former is lower than that in the latter. By contrast, China's MI is mainly based on the traditional medical literature system, and the trainees are mainly undergraduates. Therefore, the education level and faculty's strength are far behind their counterparts in Europe and the United States. Thus, we suggest that China's MI field should establish and improve the education system of medical informatics, strengthen the training of faculty by sending teachers and researchers to study abroad, introduce advanced educational concepts of medical informatics, and employ teachers from different professional backgrounds to participate in the teaching, to improve the teaching environment and curriculum selection for students. Furthermore, for the first time, we explore the differences in the themes of the papers published in the mainstream Chinese and English MI journals during the past 11 years. On the one hand, China has fully used the latecomer advantage and application advantage in hospitals, especially the extensive implementation and application of EMR [49]; on the other hand, China's MI also has no theoretical and basic research in medical data statistics and consumer health information based on the Internet and telemedicine, which requires further improvement and development.

Third, this study has extended our previous research about the academic proceedings published by main academies of MI [4, 5, 8] and further compared the countries of origin, institutions, and academic backgrounds of major authors; the academic values; and themes of articles from the MI conferences and journal articles. We have identified some important similarities and differences. (1) As for the distributions of countries of authors in the MI proceedings (AMIA Annual conference, MIE, and MedInfo), the majority of authors are distributed similarly in terms of countries of origin. The regional MI journals, including JAMIA (AMIA, USA), MIM (EFMI, Europe), and Chinese MI journals were dominated by local authors, while the international journals such as IJMI (IMIA, global) were attended by authors from various regions. (2) From the aspect of institutions and academic backgrounds, on the one hand, like the majority of affiliations in international MI proceedings, most of the authors in English-language journals (IJMI, JAMIA, MIM) and some Chinese-language journals (CJMLIS) are from universities; on the other hand, the main academic backgrounds of authors in JAMIA and MI proceedings are different from other journals, and they majored in medicine. Moreover, in terms of academic influence, journal articles are far more influential than proceedings. On the one hand, from the aspect of integrity of article search, the MI proceedings held by Chinese academies lack any stable or unified searching database and cannot guarantee the continuity of database records. In comparison, the papers published in Chinese MI journals can be completely and timely searched from mainstream Chinese databases. We think that this suggests that Chinese MI journals, as important auxiliary disciplinary systems supporting the establishment and development of MI discipline in China, yet have been valued by the academy of MI in China and their academic values have been approved to some extent. On the other hand, in terms of bibliometrics indices of papers, the average cited times and H-index of journal papers are both highly larger than those of proceedings. In terms of research contents, the themes of proceedings and journal papers are different. On the one hand, EMR is the only shared keyword in the top 10 frequent keyword lists between the two, suggesting that clinical medical informatics is the mainstream of MI research. On the other hand, the themes of proceedings are more abundant than journal articles and include some emerging themes, such as customer health informatics and infodemiology. From the practice perspective, this study proposes a method to further promote the development of disciplines in China, based on different disciplines' positioning of MI in China and Western countries. On the one hand, MI in Western countries originated from clinical practice and hospital digitization or, namely, clinical informatics; the researchers are widely distributed in the university MI systems and hospitals and broadly cooperative; and their research contents are closely linked with medical hygiene practice. The core content of the discipline of “the computer technology applied into the medical field” [50] gradually expanded to a series of subdisciplines, including public health informatics, clinical informatics, and nursing informatics. The current focus is how to process and mine the mass data generated and precipitated during medical practice and to finally advocate hygiene system infrastructure construction and clinical technology improvement, and the focus is the discovery of medical diagnosis and treatment rules and the provision of medical treatment decision-making, which suggests that the Western countries have treated MI as an applied basic discipline. On the other hand, because the MI in China originated from library science and the researchers mainly originated from medical schools, libraries, and medical informatics institutes, the cooperation among researchers was limited to institutes and rarely involved hospitals. Furthermore, the majority of the research was fueled by global trends and had no self-innovation; it did not shake off the current situation that theoretical research and clinical practice were isolated, and therefore this discipline did not develop significantly.

## 5. Limitations

Because discipline evaluation is a complicated task, we assess papers published in journals. Moreover, we use data from representative professional journals on MI in both China and Western countries but exclude all MI journals, use a limited number of searching tools to find other professional publications, and exclude achievements of patents or other forms. Furthermore, many achievements in MI may have been published in non-MI journals, but we have no perfect method or mechanism to include these papers; however, we intend to analyze these papers in subsequent studies.

## 6. Conclusions

We used bibliometrics to analyze Chinese- and English-language MI papers published in six representative MI journals between 2008 and 2018 in China, the United States, and Europe. The results of this study provide important insights for the development of MI in China and Western countries. First, compared with the Western counterparts, the number of papers published in the journals of professional associations in the field of MI in China is large and the application value is high, but the academic influence and academic value are relatively low; second, most of the authors of the Chinese papers are from hospitals, and most of the counterparts in the Western countries are from universities. The proportion of master's or doctoral degrees in the former is also lower than that of the latter; further, regarding paper themes, on the one hand, China MI has no theoretical and basic research on medical data statistics and consumer health based on the Internet and telemedicine; on the other hand, after nearly 10 years of hospital digital development, China has fully used the latecomer and application advances in hospitals and, through extensive international cooperation, has made significant advancements in and contributions to the development of medical information.

## Figures and Tables

**Table 1:** Clusters of the keywords obtained by text mining 11  years (2008–2008) of English and Chinese MI journals (IJMI, JAMIA, MIM, CDM, CJHIM, and CJMLIS) with their ten most frequent keywords.

EN-cluster 1 (26)		EN-cluster 2 (38)		EN-cluster 3 (32)		EN-cluster 4 (46)		CN-cluster 1 (75)		CN-cluster 2 (54)		CN-cluster 3 (34)	
Internet and Telemedicine		Health and Clinical Information Systems		Medical Data Statistical Analysis		EHRs and Information Management		Hospital Information Systems and EMR		Library Science and Bibliometrics Analysis		Medical Reform Policy and Health Digitization	
Keyword	*n*	Keyword	*n*	Keyword	*n*	Keyword	*n*	Keyword	*n*	Keyword	*n*	Keyword	*n*
Adult	900	Decision support system	558	Information storage and retrieval	471	Electronic health record	1568	Electronic medical record	511	University library	233	Informatization	126

Controlled study	568	User-computer interface	475	Computer program	406		966	Hospital information system	394	Information service	216	Information platform	115

Middle aged	564	Hospital information system	346	Methodology	400	United states	646	Health informatization	309	Bibliometrics	191	Health information management	114

Major clinical study	536	Physician	328	Procedure	348	Medical information system	619	The information system	193	Hospital library	153	The hospital	75

Aged	477	Hospital	307	Software	334	Information processing	555	Digital hospital	171	Medical library	133	The information center	74

Internet	450	Medical order entry system	246	Natural language processing	328	Healthcare	453	Telemedicine	169		132	Information construction	68

Questionnaire	319	Clinical practice	233	Artificial intelligence	290	Medical information	451	Healthcare	166	Medical information	127	Application	61

Telemedicine	239	Patient safety	207	Data base	274	Medical record	423	Hospital informatization	158	The database	95	Regional health	61

Utilization	227	Health personnel attitude	206	Data mining	250	Patient care	392	Medical informatization	132	Information literacy	90	Reform of the medical and health system	58

Outcome assessment	205	Practice guideline	202	Theoretical model	235	Standard	361	Information technology (IT)	130	Science and technology novelty search	86	China	57

EN: English MI journals (IJMI, JAMIA, and MIM); CN: Chinese MI journals (CDM, CJHIM, and CJMLIS).

## Data Availability

The raw data used to support the findings of this study are included in the tables and figures of the article.

## Abbreviations

MI: Medical informatics
HIT: Health information technology
CDM: China digital medicine
CJMLIS: Chinese Journal of Medical Library and Information Science
CSMI: Chinese Society of Medical Information
CJHIM: Chinese Journal of Health Informatics and Management
CMIAAS: China Medicine Information Association Annual Symposium
CHINC: China Hospital Information Network Conference
CHITEC: China Health Information Technology Exchange Conference
CPMI: China Annual Proceeding of Medical Informatics
CMIA: China Medical Informatics Association
CSMI: The Medical Informatics Branch, Chinese Medical Association
CHIA: Chinese Health Information Association
CHIMA: China Hospital Information Management Association
AMIA: American Medical Informatics Association
JAMIA: Journal of the American Medical Informatics Association
MedInfo: World Congress on Medical and Health Informatics
IMIA: International Medical Informatics Association
IJMI: International Journal of Medical Informatics
MIE: Medical Informatics Europe
MIM: Methods of Information in Medicine
EFMI: European Federation for Medical Informatics Association
CQVIP: Science and Technology Journal Database
CNKI: Chinese National Knowledge Infrastructure.

## Supplementary Materials

Supplementary Materials Table S1: research targets, basic characteristics of IJMI (IMIA, global), JAMIA (AMIA, USA), MIM (EFMI, Europe), CDM (CHIMA, China), CJHIM (CHIA, China), and CJMLIS (CSMI, China). Table S2: top 5 authors with most publications in JAMIA, IJMI, MIM, CDM, CJHIM, and CJMLIS in 2008–2018. Content S3: comparative analysis of contents of the MI academic journals in the USA, Europe, China, and the world.
