# Supplementary material for: A Comparison of the Development of Medical Informatics in China and That in Western Countries from 2008 to 2018: A Bibliometric Analysis of Official Journal Publications
Source: J Healthc Eng. 2020 Oct 12;2020:8822311. doi: 10.1155/2020/8822311 (PMC7576361; doi:10.1155/2020/8822311)
Supplement: Supplementary Materials — Table S1: research targets, basic characteristics of IJMI (IMIA, global), JAMIA (AMIA, USA), MIM (EFMI, Europe), CDM (CHIMA, China), CJHIM (CHIA, China), and CJMLIS (CSMI, China). Table S2: top 5 authors with most publications in JAMIA, IJMI, MIM, CDM, CJHIM, and CJMLIS in 2008–2018. Content S3: comparative analysis of contents of the MI academic journals in the USA, Europe, China, and the world. [file 8822311.f1.docx]

Supplementary Materials

Table S1: Research targets, basic characteristics of IJMI (IMIA-Global), JAMIA (AMIA-USA), MIM (EFMI-Europe), CDM (CHIMA- China), CJHIM (CHIA- China) and CJMLIS (CSMI - China).

| Name of journal | Time of publication | Supervising organization | Publishing organization | Publish location | Period of publication | Abstracting and Indexing | Free Access |
| --- | --- | --- | --- | --- | --- | --- | --- |
| JAMIA | 1994 | AMIA | Oxford University Press | Oxford | Bimonthly; special issue | PubMed, PubMed Central, Scopus, Medline, Embase/Excerpta Medica, SCI (2017 IF : 4.27) | Partial |
| IJMI | 1970 | IMIA and EFMI | Elsevier | Dublin | Monthly; special issue | PubMed, PubMed Central, Scopus, Medline, Embase/Excerpta Medica, Ei Compendex, SCI (2017 IF : 2.957) | Partial |
| MIM | 1962 | EFMI and German Association for Medical Informatics, Biometry and Epidemiology（GMDS） | Schattauer Publishers | Stuttgart | Bimonthly; special issue | PubMed, PubMed Central, Scopus, Medline, Embase/Excerpta Medica, SCI (2017 IF : 1.531) | Partial |
| CDM | 2007 | Ministry of Health,PRC | Committee on Information Management, Chinese Hospital Association | Beijing | Monthly; special issue | CNKI, CQVIP, Wanfang | No |
| CJHIM | 2004 | Ministry of Health,PRC | Chinese Health Information Association | Beijing | Bimonthly; special issue | CNKI, CQVIP, Wanfang | No |
| CJMLIS | 1991 | Academy of Military Medical Sciences of PLA | Medical Informatics Branch, Chinese Medical Association | Beijing | Monthly; special issue | CNKI, CQVIP, Wanfang | No |

Table S2: Top 5 authors with most publications in JAMIA, IJMI, MIM, CDM, CJHIM, and CJMLIS in 2008–2018

| Journal Name | Paper Number | First Author Number ^*^ | Corresponding Author Number | Author | Country | Affiliation | \| Educational level \|  \| \| --- \| --- \| |
| --- | --- | --- | --- | --- | --- | --- | --- | --- | --- |
| IJMI | 17 | 0 | 2 | Bates, D.W. | USA | Harvard University T.H. Chan School of Public Health | MD |
|  | 12 | 3 | 1 | Sittig, D.F. | USA | The University of Texas Health Science Center | Ph.D. |
|  | 12 | 1 | 2 | Westbrook, J.I. | Australia | Macquarie University | Ph.D. |
|  | 11 | 4 | 7 | Yu P. | Australia | University of Wollongong | Ph.D. |
|  | 10 | 1 | 4 | Jaspers, M.W.M. | Netherland | University of Amsterdam | Ph.D. |
| MIM | 20 | 4 | 1 | Haux, R. | Germany | University of Braunschweig and Hannover Medical School | Ph.D. |
|  | 16 | 6 | 1 | Ammenwerth, E. | Austria | University for Health Sciences, Medical Informatics and Technology | Ph.D., MD |
|  | 13 | 2 | 2 | Handels, H. | Germany | University Medical Center Hamburg-Eppendorf | Ph.D. |
|  | 11 | 1 | 0 | Marschollek, M. | Germany | Hannover Medical School - Wikipedia | Ph.D., MD |
|  | 10 | 0 | 1 | Gefeller, O. | Germany | University of Erlangen-Nürnberg | Ph.D. |
| JAMIA | 56 | 0 | 5 | Bates, D.W. | USA | Harvard University T.H. Chan School of Public Health | MD |
|  | 35 | 14 | 2 | Wright, A. | USA | Brigham and Women's Hospital | Ph.D. |
|  | 34 | 12 | 2 | Hripcsak, G. | USA | Columbia University Medical Center | MD, MS |
|  | 33 | 4 | 13 | Xu, H | USA | University of Texas Health Science Center at Houston | Ph.D. |
|  | 32 | 4 | 1 | Sittig, D.F. | USA | University of Texas Health Science Center at Houston | Ph.D. |
| CDM | 44 | 7 | N/A^+^ | Wanguo Xue | Beijing-China | PLA General Hospital | MS |
|  | 41 | 8 | N/A | Lirong Jia | Beijing-China | Chinese Academy of Traditional Chinese Medicine | Ph.D. |
|  | 40 | 6 | N/A | JingHua Li | Beijing-China | Chinese Academy of Traditional Chinese Medicine | MD, Ph.D. |
|  | 40 | 7 | N/A | Dan Yu | Beijing-China | Chinese Academy of Traditional Chinese Medicine | Ph.D. |
|  | 38 | 3 | N/A | Gangrong Li | Chongqing-China | First Affiliated Hospital, Army Medical University | MS |
| CJHIM | 34 | 9 | 25 | Qun Meng | Beijing-China | National Health and Family Planning Commission of the People's Republic of China | MD, Ph.D. |
|  | 24 | 3 | 7 | Yongyong Xu | Xi'an-China | Air Force Military Medical University | Ph.D. |
|  | 21 | 4 | 9 | Danhong Liu | Xi'an-China | Air Force Military Medical University | Ph.D. |
|  | 17 | 3 | 2 | Jianping Hu | Beijing-China | National Health and Family Planning Commission of the People's Republic of China | MS |
|  | 16 | 2 | 0 | Hui Wang | Beijing-China | Capital Medical University | Ph.D. |
| CJMLIS | 34 | 9 | 18 | Yan Liu | Shandong-China | Shandong Institute of Medical and Health Science Information | MD |
|  | 33 | 2 | 21 | Lei Cui | Beijing-China | China Medical University | Ph.D. |
|  | 31 | 3 | 1 | Ying Wang | Beijing-China | Medical Library of the Chinese PLA | MS |
|  | 30 | 3 | 1 | Yunxiang Du | Beijing-China | Medical Library of the Chinese PLA | BS |
|  | 27 | 4 | 5 | Rui Cheng | Beijing-China | Medical Library of the Chinese PLA | BS |

Note: * If the author is both the first author and the corresponding author, the first author is calculated by default.

+: CDM does not indicate the corresponding author.

Content S3: Comparative analysis of contents on the MI academic journals in the USA, Europe, China and the world.

## JAMIA

JAMIA as the official journal of AMIA focuses on the local MI research frontiers in the USA and its contents between 2008 and 2018 can be divided into four types, including (1) the introduction, methodology, construction technology, policy formulation, management and application of medical treatment business information systems represented by electronic admission notes; (2) data mining, machinery learning, artificial intelligence and biomedical research driven by data precipitation on electronic health records; (3) in-hospital medical decision support systems and computer-assisted clinical treatment; (4) attention, inclusion and management of patients at different age groups in clinical management and clinical trials.

The MI research in the USA is uniquely focused on three complex fields, including (1) bioinformatics: the bioinformatics is closely connected with medical informatics and links with biological data and medical research, and through bioinformatical processing (acquisition, storage, washing, analysis, management, application), aims to use the medical informatics technology and means to acquire new biological knowledge and to further derive a new interdiscipline -- biological medical informatics; (2) model algorithm research: medical informatics should be supported by informatics principles and computer methodology, and excellent models can cover a number of similar complex problems. Modeling and continual testing and perfection are powerful tools to solve medical informatics problems and obey the disciplinary definition of "computer application under medical background". (3) practical application: the final objective of medical informatics is to solve the concrete problems faced in medical practice and to facilitate the efficient circulation and utilization of medical health information. Thus, medical informatics should be tested by medical treatment practices in the real world, so as to find out problems and to solve problems by using computer methods, thereby to validate the research values of medical informatics during medical practice.

## IJMI

IJMI as the official journal of IMIA and EFMI focuses on MI research frontiers in the Western world. The topics between 2008 and 2018 can be divided into three categories, (1) the use of statistical methods to process and analyze the mass data precipitated in information systems related to medicine and health; (2) targeted at the mobile medical treatment and long-distance medical services, Internet health information diffusion, consumer health information and social media health information research, and medical informatics education; (3) The construction methodology and technology of hospital medical treatment and nursing information systems represented by electronic admission notes and electronic health records, and formulation and management of relevant policies, the auxiliary clinical decision support systems, and the artificial intelligent algorithms driven by clinical precipitation of documentary data.

The medical information research in Europe is focused on innovation and the connotations of medical informatics, including statistical methods and model algorithms. For instance, the library science and bibliometrics research in Europe often adopt the cluster analysis methods as well as the use of various artificial intelligent algorithms into data mining and pattern recognition of admission note texts. At the research perspective, the research on medical informatics in Europe covers broadly and generates various branches, such as consumer informatics, and as an important and emerging cross-sub-discipline of social behavioral science, health information psychology and medical informatics. Moreover, nurse informatics as the interdiscipline between nursing and medical informatics has been widely approved as a sub-discipline of medical informatics. As for the popularization and utilization of research achievements, the research on medical informatics in Europe is focused on the clinical practice of medical information, such as the diagnosis and treatment decision support systems under different special backgrounds, and the use of various semantic ontology and controlled words into real clinical business systems.

## MIM

MIM, as the official journal of GMDS (Germany Medical Informatics, biological measurement, epidemiology), EFMI and IMIA, focuses on MI research hotspots in Germany. The research contents between 2008 and 2018 can be divided into 3 types, including (1) technology, methods, management and application of medical treatment information systems represented by electronic admission notes and electronic health records; (2) computer-assisted signal processing and pattern recognition in clinical management and clinical trials; (3) attention, inclusion and management of different age groups in clinical management and clinical trials. Clearly, the current medical informatics research directions in Germany do not cover all areas. For instance, oral medical information has not been studied in Germany so far, and little attention has been paid to biomedical cognition, medical imaging, and medical language processing, but is focused on clinical information management, electronic admission notes, decision support systems and other basic applications.

## CDM

CDM as the official journal of CHIMA focuses on the MI research and application in China, especially hospital informatization. The relevant studies between 2008 and 2018 include (1) the introduction, construction and application of clinical information systems represented by electronic admission notes, and the focus is the roles of digital medical information construction in medical safety, medical quality and information security; (2) introduction, construction, management and application of population health informatization systems represented by electronic health records; The interoperable research on regional EHR, telemedicine, and the derived convenient medical services, such as appointed registration, payment, and long-distance consultation, and the focus is hygiene informatization and health system reform, especially the use of various hygiene management systems to improve the quality of hygiene services, reduce the medical costs, increase medical safety, establish a perfect, standardized, efficient and large-area medical treatment systems, thereby to support medical system reforms. (3) construction of hospital informatization and hospital information systems, including PACS systems, LIS systems and CDR, and the focus is the empirical lessons during the hospital informatization, and the suggestions for improvement of information systems.

## CJHIM

CJMLIS is the official journal of Chinese Health Information Association, which is the quasi-official academic institution guided by China National Health and Family Planning Commission. Thus, this journal is especially focused on the Chinese population health informatization, health care reform and hospital informatization. The main research hotspots between 2008 and 2018 can be divided into 4 types, including (1) hospital information construction in hospital information, population health information standardization and works; (2) hygiene informatization and medical system reform, and the focus is the use of various hygiene management systems to improve the hygiene insurance quality, reduce medical costs, and improve medical safety and system reform, which are also the most important issues of MI research in China; (3) introduction, construction and application of electronic health records, and the exchange and sharing of regional medical information; (4) the use of information new technology (e.g. big data, cloud computing) into clinical management, hospital management and public health management.

## CJMLIS

CJMLIS is the journal of the medical information sub-association (Chinese Medical Association), which is the first state-level professional medicine information academic organization in China. Its research hotspots between 2008 and 2018 can be divided into 3 types, including (1) medicine library information services and evidence-based medicine (including medicine libraries and university libraries to offer medical staff the pertinent, effective and innovative information services, the philosophy of library construction innovation, and the ability cultivation and comprehensive quality of library staff). This reflects that the medical informatics in China originated from medicine library informatics. Given the large number, small scales and uneven quality of medical libraries in China and since their service targets are mostly clinical medical staff and medical staff, evidence-based medicine is also an important part of information services by medical libraries. (2) literature searching and analysis, including bibliometric analysis of medical databases, paper analysis on various journals, and influence analysis of pathology research. (3) dynamics and brief news with Chinese characteristics, including academic exchanges at home and abroad, notices from various medical information academic conferences in China, and introduction into international professional medical information conferences (e.g. AMIA, EFMI, MEDINFO, HIMSS), which can be found in the introduction into information and relevant latest frontiers.
